# Intake of Hydrolyzed Casein is Associated with Reduced Body Fat Accretion and Enhanced Phase II Metabolism in Obesity Prone C57BL/6J Mice

**DOI:** 10.1371/journal.pone.0118895

**Published:** 2015-03-04

**Authors:** Morten Rahr Clausen, Xumin Zhang, Christian C. Yde, Ditte B. Ditlev, Haldis H. Lillefosse, Lise Madsen, Karsten Kristiansen, Bjørn Liaset, Hanne C. Bertram

**Affiliations:** 1 Department of Food Science, Aarhus University, Aarslev, Denmark; 2 State Key Laboratory of Genetic Engineering, School of Life Sciences, Fudan University, Shanghai, China; 3 National Institute of Nutrition and Seafood Research, Bergen, Norway; 4 Department of Biology, University of Copenhagen, Copenhagen, Denmark; IRCCS Istituto Oncologico Giovanni Paolo II, ITALY

## Abstract

The amount and form of dietary casein have been shown to affect energy metabolism and lipid accumulation in mice, but the underlying mechanisms are not fully understood. We investigated 48 hrs urinary metabolome, hepatic lipid composition and gene expression in male C57BL/6J mice fed Western diets with 16 or 32 energy% protein in the form of extensively hydrolyzed or intact casein. LC-MS based metabolomics revealed a very strong impact of casein form on the urinary metabolome. Evaluation of the discriminatory metabolites using tandem mass spectrometry indicated that intake of extensively hydrolyzed casein modulated Phase II metabolism associated with an elevated urinary excretion of glucuronic acid- and sulphate conjugated molecules, whereas glycine conjugated molecules were more abundant in urine from mice fed the intact casein diets. Despite the differences in the urinary metabolome, we observed no differences in hepatic expression of genes involved in Phase II metabolism, but it was observed that expression of *Abcc3* encoding ATP binding cassette c3 (transporter of glucuronic acid conjugates) was increased in livers of mice fed hydrolyzed casein. As glucuronic acid is derived from glucose and sulphate is derived from cysteine, our metabolomic data provided evidence for changes in carbohydrate and amino acid metabolism and we propose that this modulation of metabolism was associated with the reduced glucose and lipid levels observed in mice fed the extensively hydrolyzed casein diets.

## Introduction

It is known that intake of dietary proteins as compared to carbohydrates and lipids induces a higher thermic effect [1,2] and satiety [3–5]. Moreover, intake of high-protein diets has been associated with reduced weight gain both in rodents [6–10] and in humans [11–13]. Interestingly, several studies indicate that casein, whey protein and soy protein differ in their ability to induce weight loss [14–17]. In a randomized cross-over study the thermic effect of whey was higher than that of casein and soy protein [2]. Studies in mice have revealed that whey protein ingestion also induced lower weight gain in obesity-prone C57BL/6J mice than did casein [16–18]. Recently it was suggested that the decreased lipid accretion associated with whey intake could be explained by increased excretion of citric acid cycle intermediates in the urine [17]. Several lines of evidence therefore indicate that the choice of protein source might modulate metabolism.

In addition to protein source, protein form (i.e. intact vs hydrolyzed) might have impact on metabolism. Even though some studies report no effect [19,20], several studies report that feeding diets with hydrolyzed proteins prevented diet-induced obesity in rodents [21–26]. Others have reported lower apparent amino acid digestibility in pigs fed diets with hydrolyzed casein relative to those fed intact casein [27]. It is also likely that urea excretion increases with ingestion of hydrolyzed proteins [28,29]. Recently, we showed that, at equal energy intake, C57BL/6J mice fed Western diets containing extensively hydrolyzed casein gained less adipose tissue mass than mice fed similar diets with intact casein. Ingestion of hydrolyzed casein induced a decrease in fed plasma glucose and insulin concentrations, indicating altered carbohydrate metabolism [24]. In a follow up study, we used NMR-based metabolomics to unveil that intake of extensively hydrolyzed, as compared to intact casein, led to: i) higher fecal fat content, and lower tissue and plasma lipid levels, ii) higher branched-chain amino acid levels in feces and urine, but lower in plasma and spleen, iii) higher hepatic levels of the sulphur-containing molecules taurine and glutathione, iv) increased hepatic glycogen amount, and v) reduced levels of glucose and its metabolite lactate in feces, liver and plasma [30]. Based on these previous studies it is evident that feeding diets with extensively hydrolyzed casein modulates pathways within amino acid and carbohydrate metabolism and lipid uptake in the gastrointestinal tract in mice. However, it is still unclear how modulation of these pathways could lead to the observed divergent phenotypes.

Analysis of urinary excretion can serve as a powerful means to identify metabolic routes that are altered as a result of dietary intervention or to identify catabolic routes for specific dietary molecules. While metabolite concentrations in blood and organs, such as the liver, are dependent on metabolic status at time of sampling, urine accumulates metabolic products over time. Hence, the urinary metabolome can effectively distinguish metabolic effects that can be assigned to regulation of metabolism controlled by the dietary constituents [17,31,32].

In the present study, we investigated the metabolic changes in C57BL/6J mice induced by intake of intact or extensively hydrolyzed casein, and used LC-MS based metabolomics to study 48 hrs urine by an untargeted approach. Urinary excretion of several compounds was significantly altered and large differences between dietary treatments were observed. Intriguingly, we observed that a number of metabolites were completely absent in urine from mice fed the intact casein diets versus diets with extensively hydrolyzed casein, even though the amino acid compositions of the diets were identical. We therefore pursued identification of these compounds and report that the most significant alterations in the metabolism induced by intake of hydrolyzed casein pertain to Phase II metabolism. Mice fed diets with extensively hydrolyzed casein had higher urinary excretion of glucuronic acid- and sulphate-conjugated molecules, relative to those fed intact casein diets. By contrast, the urinary metabolome from intact casein fed mice revealed higher urinary presence of glycine-conjugated molecules in comparison to mice fed extensively hydrolyzed casein diets.

## Materials and Methods

### Dietary protein sources

Intact casein (prod #02901293) and extensively hydrolyzed casein (prod #02960138) were purchased from MP Biomedicals (Illkirch-Graffenstaden, France). The manufacturer’s web-page states that the casein hydrolysate was produced by enzymatic hydrolysis using pancreatic enzymes (not further specified). This hydrolysate had, in the form of mixed amino acids and peptides, the same amino acid composition as intact casein. The hydrolyzed casein was soluble, and about 41% of total amino acids were present as free amino acids.

### Ethics statement

Animal handling and experiments were performed in accordance with the guidelines of the Norwegian State Board of Biological Experiments with Living Animals and the board specifically approved the experiments: Norwegian approval identification No, FOTS Id: 1062 and 1840. No adverse events were observed.

### Animal study and sampling

The animal experiment and NMR-based metabolomics of organs and biofluids have been described [24,30]. Male C57BL/6J BomTac (Taconic, Denmark) mice, 8 weeks of age, were assigned to four different experimental diets; i) 16% energy from intact casein (16-I %), ii) 32% energy from intact casein (32-I %), iii) 16% energy from hydrolyzed casein (16-H %), and iv) 32% energy from hydrolyzed casein (32-H %). All diets had 35% energy from lipids composed of soybean oil (27.3 g/kg feed) and lard (144.2 g/kg feed). For further information about the experimental diets, see Lillefosse *et al*. [24]. The mice were assigned to the diets by weight to ensure both equal mean starting weight and similar group standard deviation. The mice were housed individually and maintained on a 12:12 h light-dark cycle at 28 ± 1°C. To ensure equal energy intake the amount of feed offered was determined by the group with the lowest energy intake. The mice were fed daily and body weight was recorded twice a week. After 8 weeks of feeding the mice were anesthetized with isoflurane (Isoba vet, Schering-Plough, Denmark) before they were sacrificed by cardiac puncture. In the present study mice from two experiments were included. Experimental groups consisted of 30 mice in total (n = 6–8 per group, expt. 1) and 28 mice in total (n = 7 per group, expt. 2), respectively. In both experiments growth curves followed similar patterns and several biochemical analyses consistently supported the same physiological effect of the feeding trials in both experiments [24]. During the feeding period (after 3 weeks) the mice spent 48 h in metabolic cages for urine collection (expt. 1) used for LC-MS analysis. At sacrifice (after 8 weeks) livers were harvested, weighed and freeze-clamped in liquid nitrogen and stored at -80°C (expt 1 and 2).

### LC-MS sample preparation

Prior to LC-MS analysis urine samples were diluted 5 times in acetic acid to a final acetic acid concentration of 1% and centrifuged. One hundred milligrams of each feed were suspended in 1500 μL water, carefully whirl mixed, sonicated for 15 min, and shaken for another 2 h. After extraction, 10% acetic acid was added to a final concentration of 1% acetic acid and then the samples were centrifuged 5 min at 20,000 *g*. The supernatant was filtered through Amicon Ultra 3 kDa cut-off filters (Millipore, Billerica, MA) at 14,000 *g* for 10 min.

### LC-MS analysis

LC-MS analyses of urine and feed samples were performed using a MicrOTOF-Q MS (Bruker, Bremen, Germany) coupled with an Agilent 1200 series capillary HPLC (Agilent, Santa Clara, CA) following a method reported recently [33]. The HPLC was operated at a flow rate of 12 μL/min and the mobile phases consisted of Solution A (water + 0.1% acetic acid) and Solution B (acetonitrile + 0.1% acetic acid). A double injection program was adopted to load the calibrant solution (10 mM sodium acetate) and sample individually with 2 min interval. For all analyses, 1 μL of calibrant and sample were used. A ZORBAX SB C18 column (0.5 x 150 mm, 3.5 μm, Agilent Technologies, Waldbronn, Germany) was used at 25°C for metabolite separation using the following gradient: 0% B for 2 min, linear gradient of 0–60% B in 12 min, 60–90% B in 3 min, 90% B for 2 min, 90–0% B in 1 min and 0% B for 10 min. All analyses were carried out in triplicate. MS was operated in negative ion mode, and data were collected in profile format at a frequency of 1 Hz with Active Focus mode off. The m/z range was set to 40–1000 and other optimized parameters were as follows: end plate offset, −500 V; capillary voltage, +3400 V; nebulizer pressure, 0.4 bar; dry gas flow, 4.0 L/min; dry temperature, 180°C; funnel 1 RF, 200 Vpp; funnel 2 RF, 200 Vpp; ISCID energy, 0 eV; hexapole RF, 100 Vpp; quadrupole ion energy, 5.0 eV; collision energy, 6.0 eV; collision RF 60 Vpp, transfer time, 98.4 s; pre-pulse storage, 1.0 s.

### Data preprocessing of LC-MS data

The raw data were automatically calibrated using the acetate cluster signals in DataAnalysis software (Bruker Daltonics, Bremen, Germany) and converted to MZxml files using CompassXport software (Bruker Daltonics, Bremen, Germany). The MZxml files were processed using MZmine (version 2.9.1) [34]. The detailed parameters for each file process step were as described recently [33]. For all of the metabolites discussed in the paper, the molecular formulas were further checked by SmartFormula software (Bruker Daltonics, Bremen, Germany) on the basis of high mass accuracy (< 2 mDa) and well-matched isotopic pattern (mSigma Score < 10).

The full data set after MZmine processing is available as supplemental material (S1 Table).

### Multivariate data analysis

Multivariate data analysis was performed using the SIMCA-P+ software, version 12.0.1 (Umetrics, Umeaa, Sweden). Principal component analysis (PCA) was applied to examine any intrinsic clustering of the data. Orthogonal partial least square analysis discriminant analysis (OPLS-DA) was performed to maximize the separation between classified groups of mice fed intact and hydrolyzed casein and to identify putative biomarkers. LC-MS data were mean-centered and scaled to unit-variance and cross-validated using full cross-validation. The quality of the model was determined by the goodness of prediction (Q^2^) and the fraction of Y explained by the model (R^2^Y). Furthermore, Cross Validated Analysis Of Variance (CV-ANOVA) [35] and cross-validated scores were used to test the robustness of the model. The VIP value computed by the SIMCA software was used to select variables that contributed to the discrimination between groups.

### LC-MS metabolite identification

After selection of important variables, MS^2^ experiments and database searches were applied [36–38] to characterize the discriminatory metabolites. The 50 most important LC-MS features identified by the VIP value were further investigated. Furthermore, the list of discriminatory features was matched against the result from feed analysis, and m/z values that could be detected in feed extracts were excluded from further analyses.

### Thiobarbituric acid reactive substances (TBARS)

Liver samples were homogenized and the TBARS were quantified (expt. 2) after the principle of Schmedes and Hölmer [39] with modifications as described in detail previously [40].

### Liver lipid analysis

Total lipids (expt. 2) were extracted from liver samples with chloroform: methanol, 2:1 (v:v). The lipid classes were analyzed on an automated Camaq HPTLC system and separated on HPTLC silica gel 60 F plates as previously described [41].

### Quantitative reverse transcriptase PCR (RT-qPCR)

Total RNA was purified from liver samples with TRIzol Reagent (Invitrogen, Grand Island, NY) and the quality was assessed with the NanoDrop ND-1000 UV-Vis Spectrophotometer (NanoDrop Technologies, Wilmington, DE) and the Agilent 2100 Bioanalyzer (Agilent Technologies, Santa Clara, CA). A 50 μL reverse transcription (0.5μg total RNA) reaction was performed at 48°C for 60 min utilizing a GeneAmp PCR 9700 machine (Applied Biosystems, Foster City, CA). Individual RT reactions contained 1× TaqMan RT buffer (10×), 5.5 mM MgCl_2_, 500 μM dNTP (of each), oligo dT primers (2.5 μM), 0.4 U/μL RNase inhibitor, 1.25 U/μL Multiscribe Reverse Transcriptase (N808–0234, Applied Biosystems, Foster City, CA) and RNase-free water. Quantitative reverse transcriptase PCR was run in 10 μL reactions on a LightCycler 480 Real-Time PCR System (Roche Applied Sciences, Rotkreuz, Switzerland) containing 2.0 μL cDNA and using SYBR Green Master Mix (Light Cycler 480 SYBR Green master mix kit, Roche Applied Sciences, Rotkreuz, Switzerland). Gene-specific primers were either bought (Bio-Rad Laboratories, Coralville, IA) as mouse PrimePCR SYBR green Assays, Heme oxidase 1 *(Hmox1)*, NAD(P)H dehydrogenase (quinone 1) *(Nqo1)*, Glutamate—cysteine ligase catalytic subunit *(Gclc)*, glutamate-cysteine ligase, modifier subunit *(Gclm)*, Glycine-N-acyltransferase *(Glyat);* or designed (S2 Table) using Primer Express 2.0 (Applied Biosystems, Foster City, CA). Relative gene expression was normalized to the expression of (*Tbp*, TATA box binding protein) (expt. 1 and 2).

### Statistical analysis of liver data

All data represent mean ± SE. All data sets were tested for homogeneity of variances using Levene’s test. Data were analyzed using a factorial ANOVA test with protein level (L) and protein form (F) as categorical predictors. In the case of an interaction effect between L × F, a post-hoc Fisher LSD test was performed. Data with heterogeneous variances were log-transformed before statistical analyses. P < 0.05 was considered significant. Statistics were performed with STATISTICA 9.0 (StatSoft, Tulsa, OK).

## Results and Discussion

Previously, we reported that at equal energy intake, obesity-prone C57BL/6J mice fed Western diets with extensively hydrolyzed casein after 8 weeks intervention had ~55% lower body mass gain and ~70% reduction in white adipose tissue mass, as compared to mice fed intact casein diets [24]. Concomitantly, both lower respiratory exchange ratio and reduced plasma concentrations of glucose and lactate suggested decreased carbohydrate catabolism in mice fed hydrolyzed casein diets [24]. NMR-based metabolomics indicated that fecal fat and branched-chain amino acid excretion were higher in mice given hydrolyzed casein diets, which probably could explain part, but not all, of the observed differences in the mice fed hydrolyzed vs intact casein [30]. In the present study, a LC-MS-based metabolomics analysis was performed on 48 hrs urine samples from the mice fed hydrolyzed or intact casein in order to further elucidate underlying mechanisms contributing to the observed phenotypic differences.

### Intake of extensively hydrolyzed casein had large impact on the urinary metabolome

**Fig 1:**
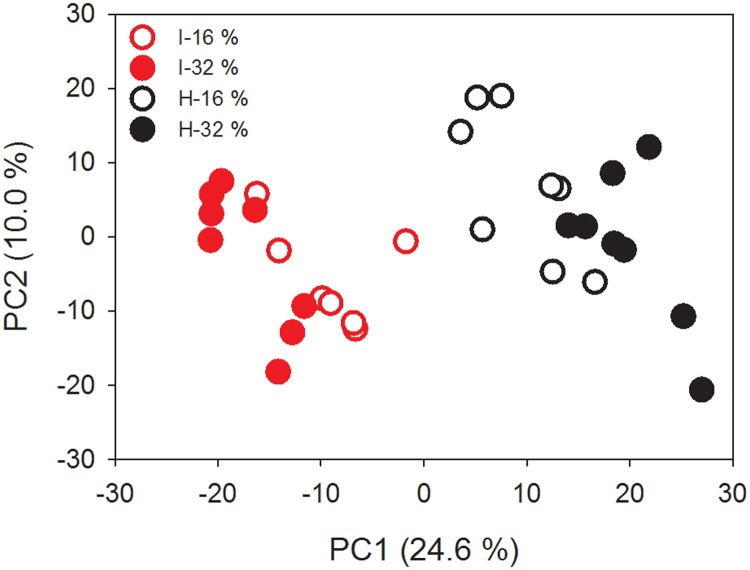
PCA score plot of urinary metabolome data. Urine from mice fed diets containing intact (I) or hydrolyzed (H) casein at 16% energy or 32% energy was analyzed using LC-MS in negative mode.

PCA was carried out on a total of 638 features detected by LC-MS in negative mode after the applied preprocessing steps had been performed. The first principal component (PC) of the PCA model accounted for 24.6% of the total variation in the data and a very clear grouping of the mice fed intact or hydrolyzed casein was observed in the score plot. A tendency to an effect of protein level (16% versus 32%) was observed; urine from mice receiving 32% protein had higher positive and negative scores on PC1 for hydrolyzed and intact casein, respectively (Fig. 1). As PCA is an unsupervised method only directed by the inherent variation of the dataset, this result confirmed that intake of extensively hydrolyzed casein had a very strong and significant impact on the metabolic response that was decisive for the global urine metabolite profile. A valid OPLS-DA model (Q^2^ = 0.88, R^2^Y = 0.91, CV-ANOVA, p = 2.6E-12) with hydrolyzed versus intact casein as the discriminant variable was constructed and an unblemished differentiation of urine samples according to dietary protein source was observed in the cross-validated score plot (S1 Fig.). Based on the VIP values computed in the OPLS-DA model, the 50 most important features for differentiating the two diets were identified (Table 1).

**Table 1:** Summary of the top 50 VIP features in urine identified from an OPLSDA model with intact versus hydrolyzed casein as discriminating variables.

VIP value	Rt	m/z	Molecular formula	Fragments, m/z	Precursors	Assignment	Effect^a^
2.00	12.30	435.1405	C_20_H_24_N_2_O_9_	259.1071; 175.0243; 113.0248; 85.0287	533.1103	Glucuronic acid and sulphate conjugate	Hydrolyzed
2.00	14.25	193.036	C_6_H_10_O_7_	131.0721; 111.0073; 87.0047		Glucuronic acid	Hydrolyzed
1.97	13.94	451.136	C_20_H_24_N_2_O_10_			435 + oxygen	Hydrolyzed
1.92	15.05	435.141	C_20_H24N2O9	241.0963; 193.0358; 175.0247; 113.0244	533.1103	Glucuronic acid and sulphate conjugate	Hydrolyzed
1.91	14.11	399.141	C21H24N2O4S	241.1208; 205.0985; 193.0356; 113.0252; 89.025		Glucuronic acid conjugate	Hydrolyzed
1.91	14.29	433.126		259.1090; 239.0794; 193.0346; 175.0275; 113.0244	643.2811	Glucuronic acid conjugate	Hydrolyzed
1.90	14.39	435.141	C20H24N2O9	193.0348; 113.0244		Glucuronic acid and sulphate conjugate	Hydrolyzed
1.88	13.04	385.125	C16H22N2O9			No MS^2^	Hydrolyzed
1.87	14.62	386.138	C16H25N3O6S			No MS^2^	Hydrolyzed
1.86	14.41	287.125	C12H20N2O6			No MS^2^	Hydrolyzed
1.86	15.05	193.036	C6H10O7	131.720		Glucuronic acid	Hydrolyzed
1.85	12.32	371.056	C14H16N2O8S			No MS^2^	Hydrolyzed
1.82	13.35	339.066	C14H16N2O6S	259.107; 181.0496; 153.0699		Sulphate conjugate	Hydrolyzed
1.80	13.43	355.061	C14H16N2O7S	275.1021; 153.0713; 121.0298; 96.9605; 79.955		Sulphate conjugate	Hydrolyzed
1.80	13.18	193.037	C6H10O7	87.0086; 85.0259	387.141	Glucuronic acid	Hydrolyzed
1.75	13.28	387.141		193.0355; 175.0254; 131.0328; 113.0247	569.1999	Glucuronic acid conjugate	Hydrolyzed
1.73	14.30	401.156	C17H26N2O9	193.048; 113.0244	401.156	Glucuronic acid conjugate	Hydrolyzed
1.70	14.10	193.036	C6H10O7			Glucuronic acid	Hydrolyzed
1.91	4.69	170.898				No MS^2^	Intact
1.80	15.17	170.082	C8H13NO3			No MS^2^	Intact
1.79	16.86	425.18	C22H25N4O5			No MS^2^	Intact
1.78	18.51	198.113	C10H17NO3	154.1253; 74.0243		Glycine conjugate	Intact
1.77	17.41	74.0241	C2H5NO2			Glycine	Intact
1.77	4.71	108.902				No MS^2^	Intact
1.76	17.03	184.098	C9H15NO3			No MS^2^	Intact
1.76	4.61	390.796				No MS^2^	Intact
1.75	12.57	323.074	C11H20N2O5S2	204.0689; 158.0641; 126.0925; 96.9576; 86.0253; 74.0070		Sulphate and glycine conjugate	Intact
1.75	18.53	199.115		74.0243		Glycine conjugate	Intact
1.74	4.63	270.843					Intact
1.74	14.46	423.144	C17H24N6O5S				Intact
1.73	16.87	186.079	C8H12NO4	74.0237		Glycine conjugate	Intact
1.72	12.71	194.045	C9H9NO4	150.0548; 100.0038; 93.0335; 74.0232		Salicyluric acid, glycine conjugate	Intact
1.72	18.37	355.139		179.1077; 164.0848; 113.0245; 87.0079			Intact
1.71	14.30	137.024	C7H6O3	93.0340		salicylic acid	Intact
1.71	14.10	408.09		296.0851; 210.0861; 143.0473; 96.959		Sulphate conjugate	Intact
2.00	11.90	226.091					Diet
1.97	11.90	225.088					Diet
1.99	12.48	226.118					Diet
1.98	14.22	241.118					Diet
1.98	12.90	227.104					Diet
1.91	14.39	275.104					Diet
1.83	11.90	243.081					Diet
1.80	11.85	243.082	C10H16N2O3S				Diet
1.75	13.26	291.097					Diet
1.72	13.14	259.076					Diet

^a^ Hydrolyzed, most abundant in urine from mice fed hydrolyzed casein; Intact, most abundant in urine from mice fed intact casein; Diet, feature detected in the diet

### Ingestion of extensively hydrolyzed casein affects the urinary excretion of phase II metabolites

While the MS instrument allows the use of very accurate masses and isotope patterns for determination of metabolite molecular formulae, identification is still obstructed by the fact that many metabolites are not available as authentic standards. The lack of standards reflects the vast number of intermediate metabolites and the diversity of metabolic reactions by which these metabolites can be transformed. This leads to a vast number of detected metabolites, of which many have not yet been identified [42]. In the present study, we have determined molecular formulae of the most important discriminatory features and used MS^2^ to identify structural units of these compounds. We observed that many compounds could be detected at several retention times, and attributed this to in-source fragmentation. As an example, a feature with an m/z of 435.140 and a molecular formula of C_20_H_24_N_2_O_9_ appeared three times in Table 1. The features with multiple appearances could be assigned to metabolites containing either glucuronic acid, sulphate, or glycine moieties (see below), but an exact molecular assignment was not possible. We assumed that such repeated features had similar chemical structures, while their precursors were different, and hence had different retention times. Several features (for instance the 435.140 peak) were found to form adducts in the mass spectrometer and therefore MS^2^ was insufficient to assign the precursor molecules. Therefore, in order to assign the precursor molecules, perfect co-elution between precursor and fragment was required (Table 1).

**Fig 2:**
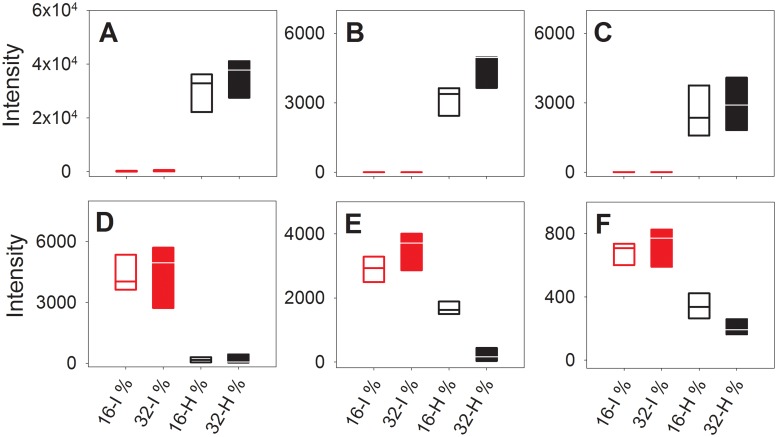
Response patterns of selected LC-MS features in urine. Urine from mice fed diets containing intact (I) or hydrolyzed (H) casein, at 16% energy or 32% energy from protein: glucuronic acid and sulphate conjugate, m/z 435.141 (A); glucuronic acid conjugate, m/z 399.141 (B); sulphate conjugate, m/z 339.066 (C); glycine conjugate, m/z 214.108 (D); glycine and sulphate conjugate, m/z 323.074 (E); salicyluric acid (a glycine conjugate, F), m/z 194.045.

Of the selected 50 discriminatory features, the majority could be assigned as molecules conjugated with either glucuronic acid (193 or 175 m/z fragments), sulphate (80 or 97 m/z fragments), or glycine (74 m/z fragment). Eighteen features were most abundant in urine from mice given the intact casein diets, whereas 18 other features were most abundant in urine from mice fed the hydrolyzed casein diets. The remaining peaks could either be detected in the diet and were possibly contaminants (10 features) or were artifacts from the sodium acetate cluster used for calibration (4 features, not shown). Tandem mass spectrometry of the discriminatory features revealed that hydrolyzed casein feeding induced excretion of glucuronic acid conjugates (11 of 18 features contained glucuronic acid) and sulphate conjugates (5 of 18 features). Interestingly, many of these compounds were completely absent in urine from mice that received intact casein (Fig. 2). Thus, the present data revealed a strong increase in the urinary excretion of D-glucuronic acid and glucuronic acid- and sulphate-conjugated molecules in mice fed hydrolyzed casein when compared to mice fed intact casein diets. Nine metabolites that were more abundant in urine from mice fed intact casein could be assigned as glycine conjugates, while two metabolites were assigned as sulphate conjugates. One of the glycine conjugates was tentatively assigned as salicyluric acid, and furthermore salicylic acid was more abundant in urine from intact casein fed mice.

D-glucuronic acid and sulphate conjugates are Phase II metabolites in mammals and are often linked to the xenobiotic metabolism of substances such as drugs, bilirubin, androgens, estrogens, mineralocorticoids, glucocorticoids, fatty acid derivatives, retinoids, and bile acids. However, while glucuronidation and sulphation improves water solubility of metabolites and thereby involves a detoxifying effect, glycine conjugation does not have the same effect on water solubility. This fact indicates that glycine conjugation may play another role than detoxification, and it was recently suggested that the function of glycine conjugation is to regulate body stores of glycine [43].

### Expression of Phase II detoxifying genes is not altered by intake of hydrolyzed casein

**Fig 3:**
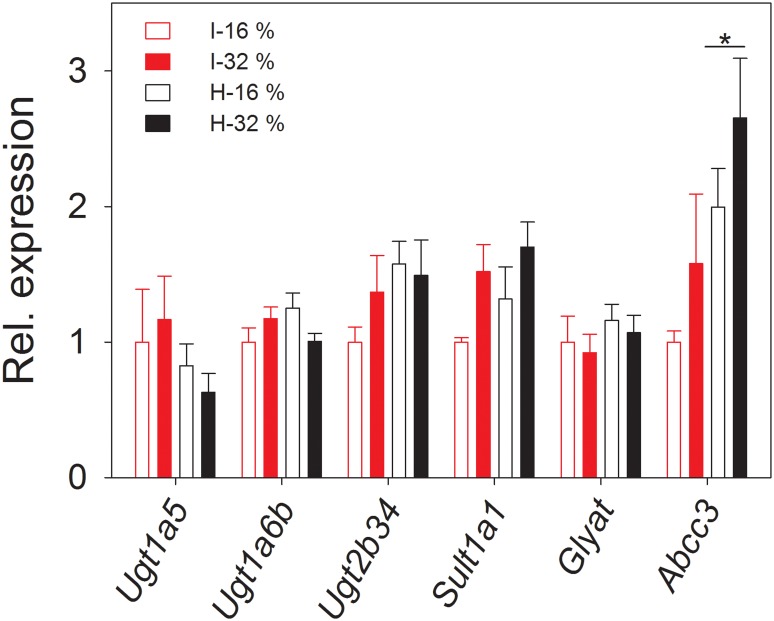
Liver gene expression related to Phase II metabolism. Gene expression in livers from mice fed diets containing intact (I) or hydrolyzed (H) casein at 16% energy or 32% energy from protein was analyzed (n = 6–8).). Gene abbreviations: *Abcc3*, ATP-binding casette, sub-family c, member 3; *Glyat*, Glycine-N-acyltransferase; *Sult1a1*, Sulfotransferase Family, Cytosolic, 1A, Phenol-Preferring, Member 1; *Ugt1a5*, UDP glucuronosyltransferase 1 family, polypeptide A5; *Ugt1a6b*, UDP glucuronosyltransferase 1 family, polypeptide A6B; *Ugt2b34*, UDP glucuronosyltransferase 2 family, polypeptide B34. * Significant effect of hydrolysis, p < 0.05.

Despite the fact that we found different excretion patterns of conjugated metabolites, there was no difference between the dietary groups in the hepatic expression of genes encoding enzymes involved in glucuronidation reactions (*Ugt1a5*, *Ugt1a6b* and *Ugt2b34*, Uridine 5′-diphospho-glucuronosyltransferase), sulphate conjugations (*Sult1a1*, sulfotransferase) and glycine conjugations (*Glyat*, glycine-N-acyltransferase) (Fig. 3). Several factors may explain this discrepancy between gene expression and the 48 hrs urinary metabolome. Firstly, the 48 hrs urinary metabolome reflects metabolic status over time, enabling us to detect minor changes that will result in phenotypic divergence over time, whereas gene expression data are measures reflecting the metabolic status of the animal at the time of sampling. Secondly, approximately one third of the ingested essential amino acids may be metabolized in the intestine mucosal cells [44], and could therefore attenuate the amino acid in-flow to the portal vein. Thirdly, genes (cytosolic sulfotransferases and uridine diphosphate-glucuronosyl transferase) that govern the conjugation are only weakly inducible [45], and may therefore not support the present/absent-behavior of the conjugates detected in the present study. In addition, elevated gene expression is not a requirement for an increase in excreted Phase II metabolites, which also could be controlled by post-translational regulation [46] or excretion in Phase III [47]. In fact, we did find elevated expression of the Phase III enzyme glucuronide transporter *Abcc3* (ATP binding cassette) [48] in mice fed hydrolyzed casein and this could contribute to the elevated level of glucuronic acid conjugates (Fig. 3). Finally, it should be noted that in our study we only measured hepatic gene expression. Phase II enzymes are expressed in most tissues and therefore it is possible that the regulation occurs in, for instance, the kidney rather than the liver [49,50].

It has previously been reported that the amount of dietary protein affects Phase I and II metabolism [51,52]. However, the exact mechanisms linking intake of hydrolyzed casein to an increased level of phase II metabolites in the urine remain unknown. Free amino acids may be absorbed directly in the intestine. Therefore, the amino acids may be taken up more rapidly and reach a higher concentration in the portal vein and liver. However, data from humans indicate that the difference between hydrolyzed and intact casein absorption is minimal and chemical alterations of the protein source are probably also necessary in order to induce Phase I and Phase II metabolism [26,53]. Other dietary perturbations have also been shown to affect Phase II metabolism using similar protein and lipid sources as in the present study; mice receiving a low fat diet had elevated RNA levels of hepatic Phase I and Phase II enzymes (*Ugt*, *Sult*) relative to mice receiving a high fat diet [54] and recently, dietary restriction was reported to induce urinary excretion of phase II metabolites and hepatic expression of Phase II enzymes in rats [55]. By contrast, mice receiving a high fat diet excreted higher amounts of glycine conjugates than mice receiving a low fat diet [56]. From the literature, it appears that sulphate and glucuronic acid conjugation are associated with intake of a low fat diet, whereas glycine conjugation is associated with intake of a high fat diet [55–57]. In the present study, despite all animals receiving the same amount of dietary fat, we found higher contents of glycine conjugates in the obese mice (fed intact casein) and higher content of sulphate and glucuronic acid conjugates in the lean mice (fed hydrolyzed casein), supporting diverging roles of these conjugation pathways with regard to body composition.

### Possible role of Phase II detoxifying reactions in glucose and cysteine metabolism

**Fig 4:**
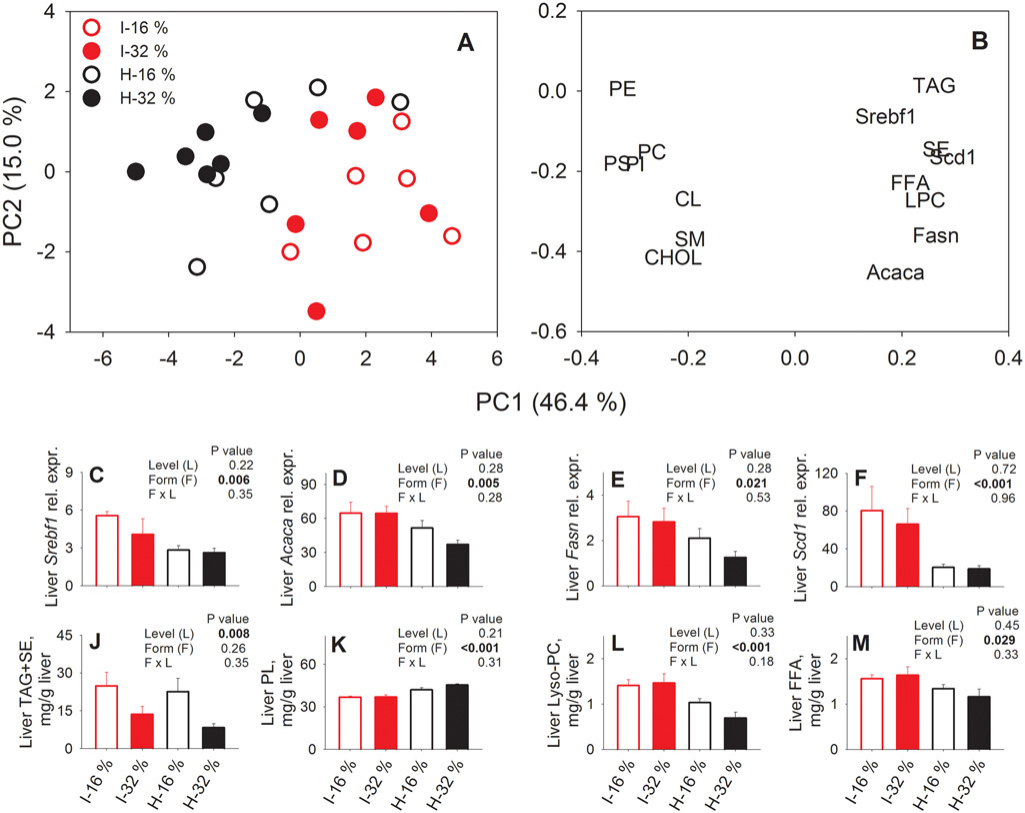
Liver lipogenic gene expression and lipid composition. PCA score plot (A) and loading plot (B) of liver genes related to lipogenesis and lipid composition data from mice fed diets containing intact (I) or hydrolyzed (H) casein, at 16% energy or 32% energy from protein (n = 5–6). Furthermore, selected data of liver gene expression (C-F) and lipid composition (J-M) are shown. These data are presented as means ± SE and p values of the effects of protein level (L), protein form (F) and the interaction (L × F) are shown in the inserts. Abbreviations: PS, phosphatidylserine; PI, phosphatidylinositol; PC, phosphatidylcholine; PE, phosphatidylethanolamine; CL, cardiolipin; SM, sphingomyelin; LPC, lyso-phosphatidylcholine; FFA, free fatty acids; Chol, cholesterol (free); SE, sterylester (sterol + fatty acid); TAG, triacylglycerol. Gene abbrevations: *Srebf1*, Sterol regulatory element binding transcription factor 1; *Acaca*, Acetyl-Coenzyme A carboxylase alpha; *Fasn*, Fatty acid synthase; *Scd1*, Stearoyl-Coenzyme A desaturase 1.

**Fig 5:**
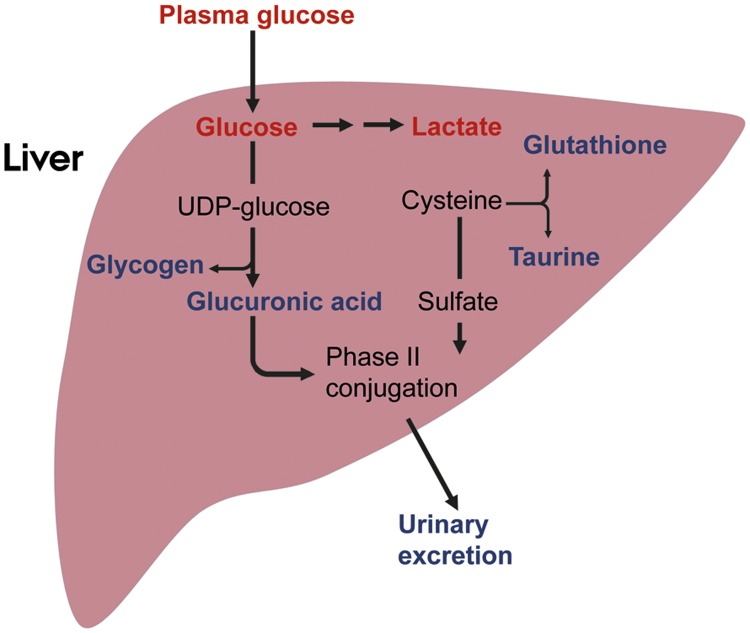
Summary of Metabolic alterations induced by ingestion of extensively hydrolyzed casein in mice. Metabolites given in red decreased after intake of hydrolyzed casein, whereas metabolites in blue increased. Mice fed diets with hydrolyzed casein have reduced plasma glucose levels [24] as well as reduced liver glucose and lactate concentrations [30]. Concomitantly, mice fed hydrolyzed casein diets exhibit a higher liver glycogen concentration [30], indicating a shift in glucose metabolism from glycolysis toward UDP-glucose synthesis, which is the intermediate substrate used both for glycogen- and D-glucuronic acid synthesis. The increased urinary abundance of D-glucuronic acid conjugated substances identified in the present study further supports such a switch in glucose metabolism by hydrolyzed casein feeding. The liver glutathione and taurine levels were shown to be increased in mice fed hydrolyzed casein [30], indicating higher hepatic cysteine availability in these mice. As cysteine is the precursor for sulphate synthesis, higher hepatic cysteine availability could explain the higher urinary content of sulphate-conjugated molecules in the hydrolyzed casein fed mice observed in the present study. In summary, these metabolic alterations induced by intake of hydrolyzed casein facilitate both the reduced plasma glucose levels and increased urinary levels of Phase II conjugated molecules.

We have previously reported that mice fed the hydrolyzed casein diet had reduced fed state plasma concentrations of glucose and insulin relative to those fed the intact casein diet [24]. Moreover, fed state plasma concentration of the glucose catabolism marker lactate was lower, which, together with a tendency toward reduced respiratory quotient during the light phase, strongly indicated less usage of glucose as an energy substrate in the mice fed hydrolyzed casein as compared to those fed intact casein diets [24]. NMR-based metabolomics confirmed reduced glucose and lactate concentrations in liver and plasma, whereas hepatic glycogen concentration was higher in mice fed the hydrolyzed casein as compared to those fed intact casein diets [30]. Together, our results strongly indicate a shift from usage of carbohydrates as energy substrate through glycolysis towards alternative metabolic usages of glucose, including storage as glycogen. Interestingly, in the body glucose can be metabolized to UDP-glucose, which subsequently can be used either in the synthesis of glycogen, or be further metabolized to D-glucuronic acid. Our LC-MS analyses in the present study suggest that more glucose was converted to D-glucuronic acid, facilitating Phase II conjugation and urinary excretion of glucuronic acid conjugated compounds. Such a redirection of glucose from glycolysis could also potentially remove substrates used in *de novo* lipogenesis. We therefore measured liver lipid contents and hepatic gene expression levels of enzymes involved in *de novo* lipogenesis. PCA scores and loadings of these data (Fig. 4A and 4B) showed that mice fed hydrolyzed casein were characterized by a decreased expression of genes involved in *de novo* lipid synthesis and decreased content of free fatty acids and triacylglycerols. As shown in Fig. 4C–4F, expression of lipogenic genes (*Srebf1* (Sterol regulatory element binding transcription factor 1), *Acaca* (Acetyl-Coenzyme A carboxylase alpha), *Fasn* (Fatty acid synthase), and *Scd1* (Stearoyl-Coenzyme A desaturase 1)) was significantly decreased after feeding with hydrolyzed casein, indicating that *de novo* lipid synthesis was repressed. Furthermore, liver free fatty acids (FFA) and lysophosphocholine were reduced in mice fed hydrolyzed casein relative to those fed intact casein diets, while a significant effect on the content of steryl esters (SE) and triacylglycerols (TAG) could not be established (Fig. 4J–4M). Therefore, we speculate that ingestion of extensively hydrolyzed casein in mice facilitates reduction of glucose concentrations by increasing glycogen deposition [30], as well as by increasing conversion of glucose to D-glucuronic acid, which subsequently can lead to decreased deposition of lipids (Fig. 5).

Sulphate is, together with glutathione and taurine, synthesized from the sulphur amino acid cysteine [58]. A major determinant of glutathione synthesis is the availability of cysteine [59], and since the level of free cysteine is kept low in the liver, it has been suggested that glutathione is a hepatic cysteine reservoir [59,60]. In our previous paper we reported that hepatic concentrations of glutathione and taurine were increased in mice fed hydrolyzed casein, indicating higher cysteine availability relative to those of mice fed intact casein [30]. If availability of cysteine was higher in hydrolyzed casein fed mice, it is also likely that more sulphate was synthesized from cysteine, which could explain the higher urinary excretion of sulphate conjugated compounds in the present study (Fig. 5).

### Hepatic Nrf-2 and p53 regulated gene expression

**Fig 6:**
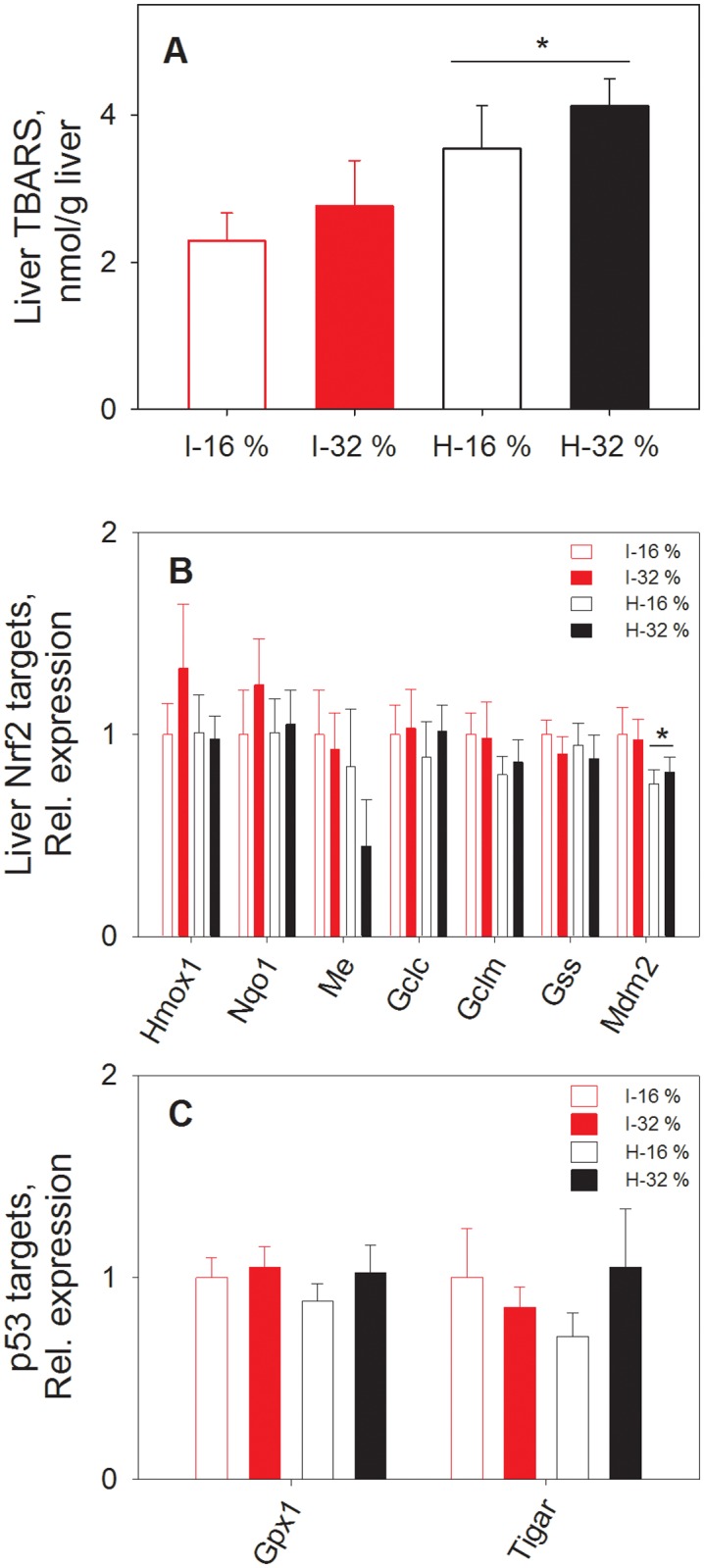
Liver TBARS and gene expression related to oxidative stress and glucose metabolism. TBARS (A), expression of Nrf-2 (B) and p53 target genes (C) in livers from mice fed diets containing intact (I) or hydrolyzed (H) casein, at 16% energy or 32% energy from protein (n = 6–8). * Significant effect of hydrolysis, p < 0.05.

Intake of extensively hydrolyzed casein diets increased fed-state plasma β-hydroxybutyrate concentration, indicating an increased hepatic mitochondrial ketogenesis in mice [24,30]. Mitochondrial respiration, including ketogenesis, is a major source of reactive oxygen species and may result in accumulation of oxidation products. We therefore measured the TBARS level in liver tissue and found a small but significantly higher level of TBARS in mice fed hydrolyzed casein compared with mice fed intact casein (Fig. 6A), indicating differences in oxidative stress between treatment groups.

Regulatory pathways that may be induced by oxidative stress include the nuclear factor erythroid 2-related factor (Nrf-2) regulated pathways [61]. Wen *et al* found that an increased level of Phase II metabolites in urine was caused by activation of the Nrf-2 pathway [55], and hence, in the present study, both increased oxidative stress and excretion of Phase II metabolites suggested that the Nrf-2 pathway was activated in mice receiving hydrolyzed casein. Furthermore, Nrf-2 has been shown to inhibit hepatic lipogenesis [62] and an induction of Nrf-2 by high fat diets has been observed [63]. Therefore, we measured expression of Nrf-2 target genes in livers from mice fed intact or hydrolyzed casein. However, no significantly different expression of genes encoding the antioxidant enzymes *Hmox1* (heme oxidase 1) or *Nqo1* (NAD(P)H dehydrogenase, quinone 1), *Me* (malic enzyme), the glutathione synthesizing enzymes *Gclc* (Glutamate—cysteine ligase catalytic subunit), *Gclm* (Glutamate—cysteine ligase modulator subunit) and *Gss* (glutathione synthase) was observed between the mice fed the different diets (Fig. 6B). However, expression of the gene encoding the negative regulator of the p53 tumor suppressor, *Mdm2* (Mouse double minute 2 homolog) was down-regulated in mice fed hydrolyzed casein (Fig. 6B), suggesting that p53 regulated pathways were modulated by hydrolyzed casein feeding. Interestingly, p53 is involved in regulation of both glucose metabolism and oxidative stress response [64]. Therefore, we measured hepatic gene expression of p53 target genes. However, we were unable to detect different expression levels of *Gpx1* (glutathione peroxidase 1) or *Tigar* (TP53-induced glycolysis and apoptosis regulator) between the dietary groups (Fig. 6C). Hence, based on hepatic gene expression analyses, we could not establish a regulatory role of neither Nrf-2 nor p53 in relation to the observed changes in Phase II metabolism, oxidative stress and lipogenesis in the present study.

## Conclusion

Intake of extensively hydrolyzed as compared to intact casein has been associated with decreased body fat accretion and reduced plasma glucose and lipid concentrations. The present study indicated that Phase II and liver lipid metabolism were significantly regulated by intake of hydrolyzed casein; the excretion pattern of Phase II conjugates and liver lipogenesis were strongly affected by protein form. Moreover, based on the present and previous studies [24,30], we speculate that the increased urinary excretion of D-glucuronic acid conjugated molecules contributed to the lower plasma and tissue glucose levels, as well as lower tissue fat accretion observed in mice fed extensively hydrolyzed casein diets. However, it is uncertain whether this contribution is quantitatively important, and further experiments are needed to confirm this.

## Supporting Information

S1 Fig Cross-validated OPLS-DA scoreplot of urinary metabolome data. Urine from mice fed diets containing intact (I) or hydrolyzed (H) casein at 16% energy or 32% energy was analyzed using LC-MS in negative mode
(DOCX)

S1 Table Raw LCMS data. (XLSX)

S2 Table Oligonucleotide probes rt-qPCR (XLSX)

## References

[1] Johnston CS, Day CS, Swan PD Postprandial thermogenesis is increased 100% on a high-protein, low-fat diet versus a high-carbohydrate, low-fat diet in healthy, young women. J Am Coll Nutr. 2002; 21: 55–61. 11838888 10.1080/07315724.2002.10719194

[2] Acheson K, Blondel-Lubrano A, Oguey-Araymon S, Beaumont M, Emady-Azar S, Ammon-Zufferey C, et al Protein choices targeting thermogenesis and metabolism. Am J Clin Nutr. 2011; 93: 525–534. 10.3945/ajcn.110.005850 21228266

[3] Potier M, Darcel N, Tomé D Protein, amino acids and the control of food intake. Curr Opin Clin Nutr Metab Care. 2009; 12: 54–58. 10.1097/MCO.0b013e32831b9e01 19057188

[4] Blouet C, Mariotti F, Azzout-Marniche D, Bos C, Mathé V, Tomé D, et al The reduced energy intake of rats fed a high-protein low-carbohydrate diet explains the lower fat deposition, but macronutrient substitution accounts for the improved. J Nutr. 2006; 136: 1849–1854. 16772448 10.1093/jn/136.7.1849

[5] Pichon L, Huneau J, Fromentin G, Tomé D A high-protein, high-fat, carbohydrate-free diet reduces energy intake, hepatic lipogenesis, and adiposity in rats. J Nutr. 2006; 136: 1256–1260. 16614413 10.1093/jn/136.5.1256

[6] Lacroix M, Gaudichon C, Martin A, Morens C, Mathé V, Tomé D, et al A long-term high-protein diet markedly reduces adipose tissue without major side effects in Wistar male rats. Am J Physiol Regul Integr Comp Physiol. 2004; 287: R934–R942. 15155276 10.1152/ajpregu.00100.2004

[7] Klaus S Increasing the protein:carbohydrate ratio in a high-fat diet delays the development of adiposity and improves glucose homeostasis in mice. J Nutr. 2005; 135: 1854–1858. 16046708 10.1093/jn/135.8.1854

[8] Ma T, Liaset B, Hao Q, Petersen RK, Fjære E, Ngo HT, et al Sucrose counteracts the anti-inflammatory effect of fish oil in adipose tissue and increases obesity development in mice. PLoS One. 2011; 6: e21647 10.1371/journal.pone.0021647 21738749 PMC3125273

[9] Madsen L, Pedersen LM, Liaset B, Ma T, Petersen RK, van den Berg S, et al cAMP-dependent signaling regulates the adipogenic effect of n-6 polyunsaturated fatty acids. J Biol Chem. 2008; 283: 7196–7205. 18070879 10.1074/jbc.M707775200

[10] Hao Q, Lillefosse HH, Fjaere E, Myrmel LS, Midtbø LK, Jarlsby RH, et al High-glycemic index carbohydrates abrogate the antiobesity effect of fish oil in mice. Am J Physiol Endocrinol Metab. 2012; 302: E1097–E1112. 10.1152/ajpendo.00524.2011 22338077

[11] Skov AR, Toubro S, Rønn B, Holm L, Astrup A Randomized trial on protein vs carbohydrate in ad libitum fat reduced diet for the treatment of obesity. Int J Obes Relat Metab Disord. 1999; 23: 528–536. 10375057 10.1038/sj.ijo.0800867

[12] Layman D, Shiue H, Sather C, Erickson D, Baum J Increased dietary protein modifies glucose and insulin homeostasis in adult women during weight loss. J Nutr. 2003; 133: 405–410. 12566475 10.1093/jn/133.2.405

[13] Foster G, Wyatt H, Hill J, McGuckin B, Brill C, Mohammed S, et al A randomized trial of a low-carbohydrate diet for obesity. N Engl J Med. 2003; 348: 2082–2090. 12761365 10.1056/NEJMoa022207

[14] Baer D, Stote K, Paul D, Harris K, Rumpler W, Clevidence B Whey protein but not soy protein supplementation alters body weight and composition in free-living overweight and obese adults. J Nutr. 2011; 141: 1–6. 10.3945/jn.110.134106 21677076 PMC3145217

[15] Takahira M, Noda K, Fukushima M, Zhang B, Mitsutake R, Uehara Y, et al Randomized, Double-Blind, Controlled, Comparative Trial of Formula Food Containing Soy Protein vs. Milk Protein in Visceral Fat Obesity. Circ J. 2011; 75: 2235–2243. 21768737 10.1253/circj.cj-10-1013

[16] Tranberg B, Hellgren LI, Lykkesfeldt J, Sejrsen K, Jeamet A, Rune I, et al Whey protein reduces early life weight gain in mice fed a high-fat diet. PLoS One. 2013; 8: e71439 10.1371/journal.pone.0071439 23940754 PMC3735523

[17] Lillefosse H, Clausen M, Yde CC, Ditlev DB, Zhang X, Du Z-Y, et al Loss of Tricarboxylic Acid Cycle Intermediates as Revealed by Metabolomics Studies—An Underlying Mechanism to Reduce Lipid Accretion by Whey Protein Ingestion. J Proteome Res. 2014; 13: 2560–2570. 10.1021/pr500039t 24702026 PMC4045150

[18] Shi J, Tauriainen E, Martonen E, Finckenberg P, Ahlroos-Lehmus A, Tuomainen A, et al Whey protein isolate protects against diet-induced obesity and fatty liver formation. Int Dairy J. 2011; 21: 513–522.

[19] Aoyama T, Fukui K, Takamatsu K, Hashimoto Y, Yamamoto T Soy protein isolate and its hydrolysate reduce body fat of dietary obese rats and genetically obese mice (yellow KK). Nutrition. 2000; 16: 349–354. 10793303 10.1016/s0899-9007(00)00230-6

[20] Itoh H, Kishi T, Chibata I Comparative effects of casein and amino acid mixture simulating casein on growth and food intake in rats. J Nutr. 1973; 103: 1709–1715. 4752973 10.1093/jn/103.12.1709

[21] Kim KM, Chang UJ, Kang DH, Kim JM, Choi YM, Suh HJ Yeast hydrolysate reduces body fat of dietary obese rats. Phytother Res. 2004; 18: 950–953. 15597316 10.1002/ptr.1582

[22] Broccali G, Berti M, Pistolesi E, Cestaro B Hydrolyzed milk-serum peptides reduce body weight and fat content of dietary obese rats ameliorating their antioxidant status and liver functions. Panminerva Med. 2005; 47: 123–129. 16210997

[23] Liaset B, Madsen L, Hao Q, Criales G, Mellgren G, Marschall H-U, et al Fish protein hydrolysate elevates plasma bile acids and reduces visceral adipose tissue mass in rats. Biochim Biophys Acta. 2009; 1791: 254–262. 10.1016/j.bbalip.2009.01.016 19416649

[24] Lillefosse HH, Tastesen HS, Du Z, Ditlev D, Thorsen F, Madsen L, et al Hydrolyzed Casein Reduces Diet-Induced Obesity in Male C57BL/6J Mice. J Nutr. 2013; 143: 1367–1375. 10.3945/jn.112.170415 23843475

[25] Liaset B, Hao Q, Jørgensen H, Hallenborg P, Du Z-Y, Ma T, et al Nutritional regulation of bile acid metabolism is associated with improved pathological characteristics of the metabolic syndrome. J Biol Chem. 2011; 286: 28382–28395. 10.1074/jbc.M111.234732 21680746 PMC3151081

[26] Stanstrup J, Rasmussen JE, Ritz C, Holmer-Jensen J, Hermansen K, Dragsted LO Intakes of whey protein hydrolysate and whole whey proteins are discriminated by LC—MS metabolomics. Metabolomics. 2013; Available: http://link.springer.com/10.1007/s11306-013-0607-9. Accessed 17 January 2014.

[27] Yin Y, Huang R, Libao-Mercado A, Jeaurond E, de Lange C, Rademacher M Effect of including purified jack bean lectin in casein or hydrolysed casein-based diets on apparent and true ileal amino acid digestibility in the growing pig. Anim Sci. 2004; 79: 283–291.

[28] Lacroix M, Bos C, Léonil J, Airinei G, Luengo C, Daré S, et al Compared with casein or total milk protein, digestion of milk soluble proteins is too rapid to sustain the anabolic postprandial amino acid requirement. Am J Clin Nutr. 2006; 84: 1070–1079. 17093159 10.1093/ajcn/84.5.1070

[29] Rérat A Nutritional supply of proteins and absorption of their hydrolysis products: consequences on metabolism. Proc Nutr Soc. 1993; 52: 335–344. 8234355 10.1079/pns19930069

[30] Yde CC, Clausen MR, Ditlev DB, Lillefosse H, Madsen L, Kristiansen K, et al. Multi-block PCA and multi-compartmental study of the metabolic responses to intake of hydrolysed versus intact casein in C57BL/6J mice by NMR-based metabolomics. Metabolomics. 2014; Available: http://link.springer.com/10.1007/s11306-014-0623-4. Accessed 30 January 2014.

[31] Kristensen M, Engelsen SB, Dragsted LO LC—MS metabolomics top-down approach reveals new exposure and effect biomarkers of apple and apple-pectin intake. Metabolomics. 2011; 8: 64–73.

[32] Yde CC, Westerhuis J a, Bertram HC, Bach Knudsen KE Application of NMR-based metabonomics suggests a relationship between betaine absorption and elevated creatine plasma concentrations in catheterised sows. Br J Nutr. 2012; 107: 1603–1615. 10.1017/S0007114511004909 22673149

[33] Zhang X, Clausen MR, Zhao X, Zheng H, Bertram HC Enhancing the power of liquid chromatography-mass spectrometry- based urine metabolomics in negative ion mode by optimization of the additive. Anal Chem. 2012; 84: 7785–7792. 10.1021/ac3013835 22888765

[34] Pluskal T, Castillo S, Villar-Briones A, Oresic M MZmine 2: Modular framework for processing, visualizing, and analyzing mass spectrometry-based molecular profile data. BMC Bioinformatics. 2010; 11: 395 10.1186/1471-2105-11-395 20650010 PMC2918584

[35] Eriksson L, Trygg J, Wold S CV-ANOVA for significance testing of PLS and OPLS—models. J Chemom. 2008; 22: 594–600.

[36] Wishart DS, Knox C, Guo AC, Eisner R, Young N, Gautam B, et al HMDB: a knowledgebase for the human metabolome. Nucleic Acids Res. 2009; 37: D603–D610. 10.1093/nar/gkn810 18953024 PMC2686599

[37] Smith CA, O’Maille G, Want EJ, Qin C, Trauger SA, Brandon TR, et al METLIN—A metabolite mass spectral database. Ther Drug Monit. 2005; 27: 747–751. 16404815 10.1097/01.ftd.0000179845.53213.39

[38] Wishart DS, Jewison T, Guo AC, Wilson M, Knox C, Liu Y, et al HMDB 3.0—The Human Metabolome Database in 2013. Nucleic Acids Res. 2013; 41: D801–D807. 10.1093/nar/gks1065 23161693 PMC3531200

[39] Schmedes A, Hølmer G A new thiobarbituric acid (TBA) method for determining free malondialdehyde (MDA) and hydroperoxides selectively as a measure of lipid peroxidation. J Am Oil Chem Soc. 1989; 6: 813–817.

[40] Hamre K, Næss T, Espe M, Holm J, Lie Ø A formulated diet for Atlantic halibut (Hippoglossus hippoglossus, L.) larvae. Aquac Nutr. 2001; 7: 123–132.

[41] Liaset B, Julshamn K, Espe M Chemical composition and theoretical nutritional evaluation of the produced fractions from enzymic hydrolysis of salmon frames with Protamex^TM^ . Process Biochem. 2003; 38: 1747–1759.

[42] Roux A, Xu Y, Heilier J-F, Olivier M-F, Ezan E, Tabet J-C, et al Annotation of the human adult urinary metabolome and metabolite identification using ultra high performance liquid chromatography coupled to a linear quadrupole ion trap-Orbitrap mass spectrometer. Anal Chem. 2012; 84: 6429–6437. 10.1021/ac300829f 22770225

[43] Beyoğlu D, Smith RL, Idle JR Dog bites man or man bites dog? The enigma of the amino acid conjugations. Biochem Pharmacol. 2012; 83: 1331–1339. 10.1016/j.bcp.2011.12.031 22227274 PMC3314100

[44] Stoll B, Henry J, Reeds PJ, Yu H, Jahoor F, Burrin DG Catabolism dominates the first-pass intestinal metabolism of dietary essential amino acids in milk protein-fed piglets. J Nutr. 1998; 128: 606–614. 9482771 10.1093/jn/128.3.606

[45] Zamek-Gliszczynski MJ, Hoffmaster K a, Nezasa K, Tallman MN, Brouwer KLR Integration of hepatic drug transporters and phase II metabolizing enzymes: mechanisms of hepatic excretion of sulfate, glucuronide, and glutathione metabolites. Eur J Pharm Sci. 2006; 27: 447–486. 16472997 10.1016/j.ejps.2005.12.007

[46] Basu NK, Kole L, Owens IS Evidence for phosphorylation requirement for human bilirubin UDP-glucuronosyltransferase (UGT1A1) activity. Biochem Biophys Res Commun. 2003; 303: 98–104. 12646172 10.1016/s0006-291x(03)00241-9

[47] Xu C, Li CY-T, Kong A-NT Induction of phase I, II and III drug metabolism/transport by xenobiotics. Arch Pharm Res. 2005; 28: 249–268. 15832810 10.1007/BF02977789

[48] Geier A, Wagner M, Dietrich CG, Trauner M Principles of hepatic organic anion transporter regulation during cholestasis, inflammation and liver regeneration. Biochim Biophys Acta. 2007; 1773: 283–308. Available: http://www.ncbi.nlm.nih.gov/pubmed/17291602. Accessed 6 June 2014. 17291602 10.1016/j.bbamcr.2006.04.014

[49] Dooley TP, Haldeman-Cahill R, Joiner J, Wilborn TW Expression profiling of human sulfotransferase and sulfatase gene superfamilies in epithelial tissues and cultured cells. Biochem Biophys Res Commun. 2000; 277: 236–245. Available: http://www.ncbi.nlm.nih.gov/pubmed/11027669. Accessed 17 December 2014. 11027669 10.1006/bbrc.2000.3643

[50] Shelby M, Cherrington N, NR V, CD K Tissue mRNA expression of the rat UDP-glucuronosyltransferase gene family. Drug Metab Dispos. 2003; 31: 326–333. Available: http://dmd.aspetjournals.org/content/31/3/326.short. Accessed 17 December 2014. 12584160 10.1124/dmd.31.3.326

[51] Butler LE, Dauterman WC The Effect of Dietary Protein Levels on Xenobiotic Biotransformations in F344 Male Rats. Toxicol Appl Pharmacol. 1988; 95: 301–310. 3420617 10.1016/0041-008x(88)90166-4

[52] Woodall GM, Dauterman WC, DeMarini DM Effect of dietary casein levels on activation of promutagens in the spiral Salmonella mutagenicity assay. II. Studies with induced rat liver S9. Mutat Res. 1996; 360: 127–143. 8649465 10.1016/0165-1161(96)00006-4

[53] Calbet JAL, Holst JJ Gastric emptying, gastric secretion and enterogastrone response after administration of milk proteins or their peptide hydrolysates in humans. Eur J Nutr. 2004; 43: 127–139. 15168035 10.1007/s00394-004-0448-4

[54] Ghose R, Omoluabi O, Gandhi A, Shah P, Strohacker K, Carpenter KC, et al Role of high-fat diet in regulation of gene expression of drug metabolizing enzymes and transporters. Life Sci. 2011; 89: 57–64. Available: http://www.pubmedcentral.nih.gov/articlerender.fcgi?artid=3156825&tool=pmcentrez&rendertype=abstract. Accessed 18 December 2013. 10.1016/j.lfs.2011.05.005 21620874 PMC3156825

[55] Wen H, Yang H-J, An YJ, Kim JM, Lee DH, Jin X, et al Enhanced phase II detoxification contributes to beneficial effects of dietary restriction as revealed by multi-platform metabolomics studies. Mol Cell Proteomics. 2013; 12: 575–586. 10.1074/mcp.M112.021352 23230277 PMC3591652

[56] Boulangé C, Claus S, Chou C, Collino S, Montoliu I, Kochhar S, et al Early Metabolic Adaptation in C57BL/6 Mice Resistant to High Fat Diet Induced Weight Gain Involves an Activation of Mitochondrial Oxidative Pathways. J Proteome Res. 2013; 12: 1956–1968. 10.1021/pr400051s 23473242

[57] Köhle C, Bock KW Coordinate regulation of Phase I and II xenobiotic metabolisms by the Ah receptor and Nrf2. Biochem Pharmacol. 2007; 73: 1853–1862. 17266942 10.1016/j.bcp.2007.01.009

[58] Stipanuk M, Dominy J, Lee J-I, Coloso R Mammalian cysteine metabolism: new insights into regulation of cysteine metabolism. J Nutr. 2006; 136: 1652–1659. 16702335 10.1093/jn/136.6.1652S

[59] Lu SC Regulation of hepatic glutathione synthesis: current concepts and controversies. FASEB J. 1999; 13: 1169–1183. 10385608

[60] Tateishi N, Higashi T, Naruse A, Nakashima K, Shiozaki H, Sakamoto Y Rat liver glutathione: possible role as a reservoir of cysteine. J Nutr. 1977; 107: 51–60. 13167 10.1093/jn/107.1.51

[61] Motohashi H, Yamamoto M Nrf2-Keap1 defines a physiologically important stress response mechanism. Trends Mol Med. 2004; 10: 549–557. 15519281 10.1016/j.molmed.2004.09.003

[62] Kay HY, Kim WD, Hwang SJ, Choi H-S, Gilroy RK, Wan Y-JY, et al Nrf2 inhibits LXRα-dependent hepatic lipogenesis by competing with FXR for acetylase binding. Antioxid Redox Signal. 2011; 15: 2135–2146. 10.1089/ars.2010.3834 21504366 PMC6468953

[63] Vomhof-Dekrey EE, Picklo MJ The Nrf2-antioxidant response element pathway: a target for regulating energy metabolism. J Nutr Biochem. 2012; 23: 1201–1206. 10.1016/j.jnutbio.2012.03.005 22819548

[64] Bensaad K, Vousden KH P53: New Roles in Metabolism. Trends Cell Biol. 2007; 17: 286–291. 17481900 10.1016/j.tcb.2007.04.004

