# Supplementary figures and images for: Intake of Hydrolyzed Casein is Associated with Reduced Body Fat Accretion and Enhanced Phase II Metabolism in Obesity Prone C57BL/6J Mice
Source: PLoS One. 2015 Mar 4;10(3):e0118895. doi: 10.1371/journal.pone.0118895 (PMC4349863; doi:10.1371/journal.pone.0118895)

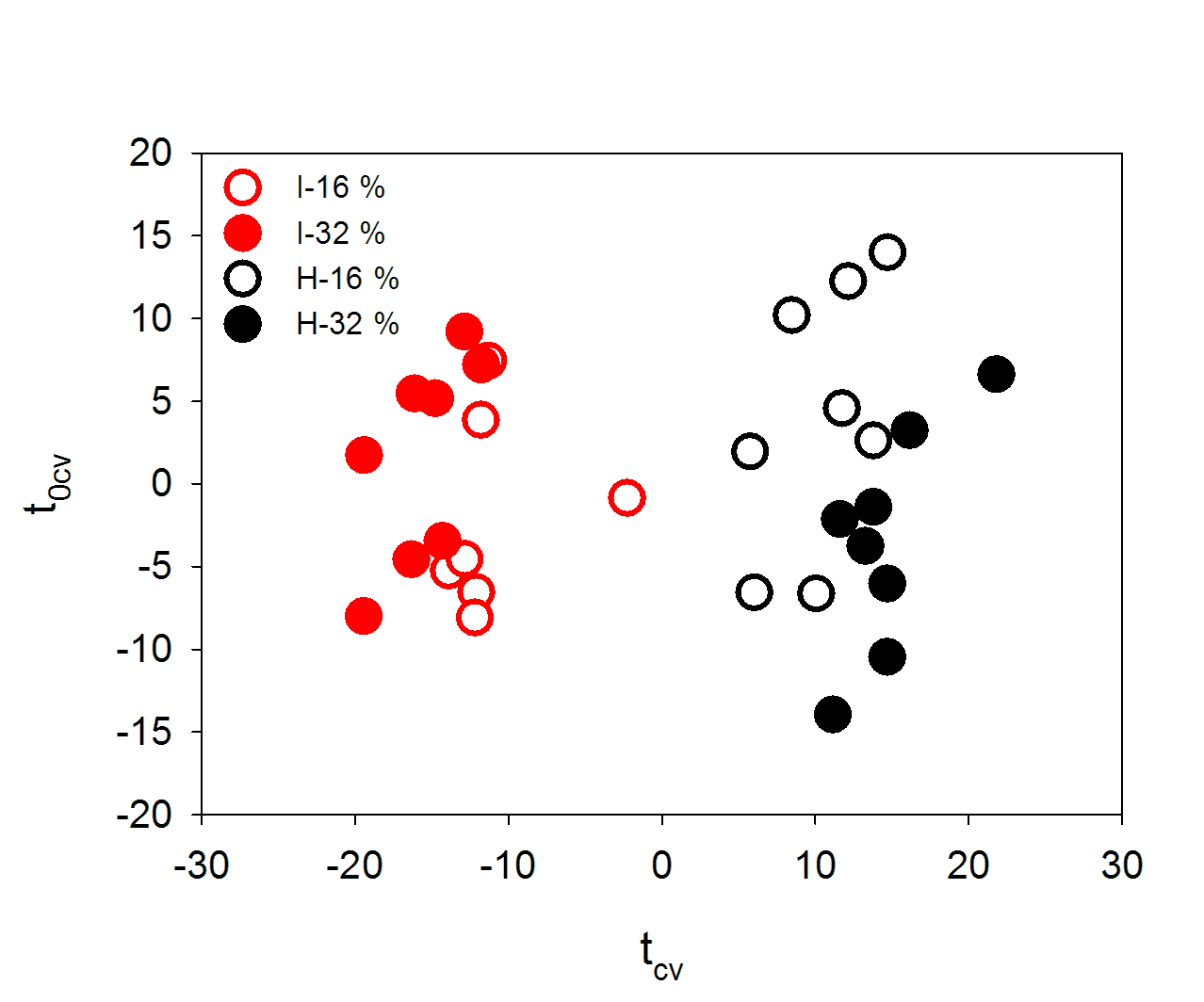

Supplement: S1 Fig — Urine from mice fed diets containing intact (I) or hydrolyzed (H) casein at 16% energy or 32% energy was analyzed using LC-MS in negative mode (DOCX) [file pone.0118895.s001.docx]
